# Quantum-dot sensitized hierarchical NiO p–n heterojunction for effective photocatalytic performance†

**DOI:** 10.1039/d2ra05657g

**Published:** 2022-11-11

**Authors:** Junaid Khan, Gohar Ali, Ayesha Samreen, Shahbaz Ahmad, Sarfraz Ahmad, Mehmet Egilmez, Sadiq Amin, Nadia Khan

**Affiliations:** Department of Physics, University of Peshawar Peshawar Pakistan; Department of Materials Science and Chemical Engineering, Hanyang University Ansan 15588 Republic of Korea; Department of Physics, American University of Sharjah Sharjah POBOX: 26666 United Arab Emirates megilmez@aus.edu; Materials Science and Engineering Program, College of Arts and Sciences, American University of Sharjah Sharjah POBOX: 26666 United Arab Emirates; Department of Mathematics, Abbottabad University of Science and Technology Abbottabad 22500 Pakistan; Material Research Laboratory, Department of Physics, University of Peshawar 25120 Pakistan; Department of Physics, Khushal Khan Khattak University Karak 27200 Khyber-Pakhtunkhwa Pakistan

## Abstract

A facile and low-cost pseudo successive ionic layer adsorption and reaction technique was used to deposit cadmium sulfide quantum dots (CdS QDs) on hierarchical nanoflower NiO to form an effective and intimate NiO/CdS p–n heterojunction system. The synthesized hierarchical p–n heterojunctions demonstrated effective photocatalytic activity due to the enhanced separation and transport of photogenerated charge carriers compared to standalone NiO. The dye degradation efficiency of optimized CdS QDs that form p–n heterojunctions was examined by rhodamine B and methylene blue dyes under UV-vis irradiation. The improved photocatalytic performance can be accredited to a large morphological surface, and the successful deposition of CdS QDs to form an active p–n junction for efficient charge separation and migration. The morphological, structural, optical, charge transfer and photocatalytic characteristics of synthesized hierarchical p–n junction photocatalyst were studied by scanning electron microscopy, UV-visible absorbance, X-ray diffraction, photoluminescence spectroscopy, electrochemical spectroscopy, and Fourier transform infrared spectra. Additionally, scavenging experiments were performed to find out the energetic species taking part in dye-degradation, and a rational reaction mechanism has been proposed.

## Abstract 2

A facile and low-cost pseudo successive ionic layer adsorption and reaction technique was used to deposit cadmium sulfide quantum dots (CdS QDs) on hierarchical nanoflower NiO to form effective and intimate NiO/CdS, p–n heterojunctions.

## Graphical abstract

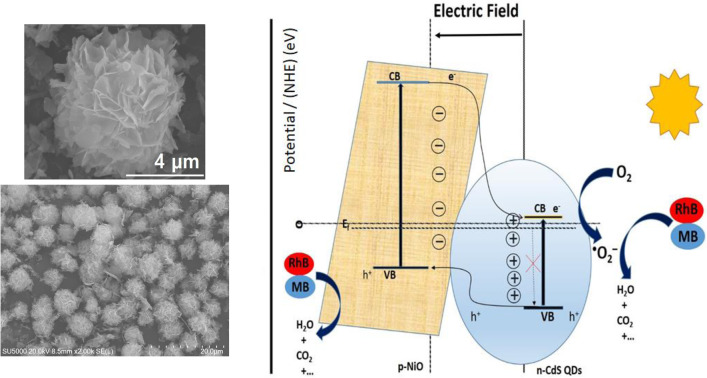

## 1. Introduction

The expansion in the utilization of various types of organic dyes in certain industries such as textile and cosmetics rationally increased the demand for water purification to reduce the hazardous impacts on the environment and health.^1,2^ In order to account for the problem, several materials have been synthesized for the effective degradation of organic dyes, such as polymeric bio adsorbents,^3^ multilamellar mesoporous nanocomposites,^4^ carbohydrate polymeric sustainable adsorbents,^5^ encapsulation nanocomposites,^6^ sensitivity enhanced nanocomposites,^7^ nanosized metal oxides,^8^*para*-aminobenzoic acid modified activated carbon,^9^ nonporous aluminosilicate monoliths,^10^ n–p–n heterojunctions,^11^ and several mechanistic approaches.^12^ The utilization of all these materials effectively degrades the dye molecules. Furthermore, it is well known that the morphology, and surface area of the photocatalyst play a significant role in the photocatalytic performance to degrade dye molecules efficiently.^13^ 3D materials such as hierarchical nanoflower provide more coordination to the Ni atoms (in the context of NiO), oxygen vacancies, as well as more defect sites on the surface leading to large morphological surfaces. They will have larger surface areas compared to 1-D and 2D materials such as nanoparticles, nanorods, nanosheets, nanospheres, nanotubes, *etc.* Large surface area characteristics play a vital role in dye degradation.^13,14^

Hence, the conversion of low-dimensional nanostructures (0D, 1D, 2D) into three-dimensional (3D) superstructures acquires great attention because of their unique physical and chemical characteristics compared with low-dimensional nanostructures.^15,16^ The applications-driven optical, photocatalytic, and electronic properties of nanomaterials are directly related to their morphology. In particular, a wide range of tunability of such functionalities has been obtained through developing various morphologies such as nanosheets,^17^ nanorods,^18^ hollow spheres,^19^*etc.* These synthesized nanostructural materials are widely used in photocatalysis, solar cell, and battery applications.^20,21^ Among many transition metal oxides, nickel oxide (NiO) is gaining significant attention nowadays due to its applicability in many fields such as fuel-cell research,^18^ catalysis,^22^ gas sensing,^23^ magnetic studies,^24,25^*etc.* Senobari *et al.*, synthesized p–n heterojunction NiO–CdS nanoparticles degrading 82% MB dye. Similarly, Deng *et al.*, synthesized 1D hierarchical CdS NPs/NiO NFs heterostructures with enhanced photocatalytic activity degrading 91.2% Congo red dye.^2,26^ Meng *et al.*, studied the degradation mode as well as features of different pesticides *via* photolysis and hydrolysis techniques and found that the decomposition or separation of molecules by the irradiation of light through the photolysis process is critically important for the applicability of a photocatalytic material.^27,28^ Lin *et al.*, studied the performance of the well-dispersed CdS–NiO heterostructure under UV-visible and visible light irradiation and found excellent characteristics.^29^

To date, several morphologies of NiO have been synthesized by different groups *e.g.*, Beach *et al.*^30^ prepared NiO microspheres *via* a solvothermal technique. Similarly, Zeng *et al.*^31^ and Chen *et al.*^32^ synthesized hollow and flowerlike NiO nanostructures respectively. Among these synthesized nanostructures flowerlike NiO possesses large coordinate unsaturated Ni atoms, large oxygen vacancies, and more defect sites on the surface of NiO, making flowerlike NiO more attractive for catalytic reactions. However, pristine NiO is not suitable for the photodegradation process due to its large bandgap (∼3.4 eV) and prompt charge recombination rate. To overcome these limitations and to effectively separate charges, the photogenerated charge carrier p–n junction formation method can be applied.^33^ For that purpose, n-type material has been frequently used. Recently, cadmium sulfide (CdS) due to its distinguished characteristics such as visible light bandgap at room temperature, and sufficiently negative flat-band potential has been suggested as a suitable candidate to form p–n junction with NiO for effective charge separation and transport.^29,34^ In CdS, the excitons Bohr radius is approximately 2.85 nm which makes this material a promising material for quantum dots. When the size of the CdS particles approaches to excitons Bohr radius along with increased surface-to-volume ratio, CdS exhibits excellent nonlinear optical properties which in return give rise to rich luminescent properties. Furthermore, the transport of photogenerated charge carriers from CdS enhances its resistance against photo corrosion thus improving the stability and reliability in different applications.^35,36^ For example, Lin *et al.*, synthesized the CdS–NiO heterostructure *via* the hydrothermal method demonstrating enhanced hydrogen evolution performance compare to pristine NiO and CdS.^29^ Moreover, Deng *et al.* synthesized 1D CdS/NiO heterostructure by using a chemical bath technique demonstrating enhanced visible-light photocatalytic activity.^2^ Such study motivated us to fabricate the CdS quantum dot (QDs) sensitized hierarchical NiO p–n heterojunction *via* pseudo-successive ionic layer adsorption and reaction (p-SILAR) technique. In the present work, we synthesized NiO/CdS p–n heterojunction for the first time by depositing an optimum amount of CdS QDs on hierarchical NiO *via* the p-SILAR technique. Which is a facile, simple, and cost-effective technique for the successful formation as well as deposition of CdS QDs compared to other methods. On one hand, the synthesized nanocomposite enhances the absorbance of pristine NiO while on the contrary enhancing photo corrosion resistance of CdS QDs thus improving the stability, reusability, and performance. The improved photocatalytic performance can be accredited to the large surface area, a large number of unsaturated vacancies present on the surface acting as a reaction site, as well as optimum deposition of QDs to form an effective p–n heterojunction for effective charge separation and migration. The synthesized p–n heterojunction offers stability, reusability as well as cost-effectiveness.

## 2. Experimental details

### 2.1. Synthesis of NiO hierarchical nanoflower

The NiO hierarchical nanoflower structures were synthesized by the hydrothermal method. Simply 0.5 M solution of nickel nitrate Ni(NO_3_)_2_ made in deionized (D.I) water and a specific quantity of ethylene diamine (3.2 mL) added dropwise to it under continuous stirring. Then 7 M solution of NaOH in D.I water was poured into the above solution and stirred for 30 minutes. Next, the prepared solution was transferred to an autoclave keeping the temperature at 150 °C for 4 h to synthesize the Ni(OH)_2_. The synthesized Ni(OH)_2_ was annealed at 400 °C for 300 minutes to obtain the NiO hierarchical nanoflower.

### 2.2. Synthesis of NiO/CdS p–n hierarchical heterojunction

Before the p-SILAR process, anionic and cationic precursors were synthesized by the method reported by Naeem *et al.*^14^ For CdS QDs deposition we have prepared 0.05 M solution of Cd (NO_3_)_2_·4H_2_O in methanol as a cationic precursor as well as 0.05 M solution of Na_2_S·5H_2_O in D.I water and methanol keeping 1 : 1 ratio to make sure it is acting as an anionic precursor.^13,14^ In order to prepare hierarchical NiO/CdS QDs p–n heterojunction we utilized already optimized recipes^14^ (0.15 g of NiO powder) into the centrifuge tube and poured with 20 mL of cationic precursor.^13,14,37^ Then it was centrifuged at 8000 revolutions per minute (rpm) for 5 minutes. Later, 20 mL of methanol was poured to remove loosely attached Cd-ions. Then 0.05 M S solution acting as an anionic precursor was added and centrifuged at 8000 rpm for 5 minutes. Rinsing was repeated once more time (20 mL of methanol) to eliminate additional S-ions. Different numbers of QDs cycles were deposited on NiO to make an effective p–n heterojunction by using this p-SILAR technique.^37^

### 2.3. Structural and spectroscopical characterization

X-Ray diffraction (XRD) patterns were obtained using an X-ray diffractometer (D/Max-2500/PC, Rigaku) to study the phase purity and crystallinity of synthesized ternary nanocomposite. A scanning electron microscope (FESEM, HITACHI, S4800) coupled with energy-dispersive X-ray spectroscopy (EDS) was utilized to study the morphological details as well as to acquire associated compositional information. PL spectra were acquired using HORIBA-Lab RAMHR to examine the recombination rate of the photogenerated charges carrier. Electrochemical impedance spectra were obtained using (CHI 760 D, CH Instruments) within 100 Hz–1 MHz frequency range with a constant bias of 0.2 V. UV-vis study was performed on a UV-vis spectrophotometer (JASCO, V-650) using BaSO_4_ as a reference. Fourier transforms infrared (FT-IR) data were obtained by an FT-IR spectrometer (Thermo Fisher Scientific, NICOLET iS10).

### 2.4. Photocatalytic performance evaluation

The photocatalytic performance of the synthesized samples was estimated for dye degradation of rhodamine B (RhB) as well as methylene blue (MB).^38,39^ Initially, 72 mg of RhB and 60 mg of MB were dispersed separately in 1000 mL of fresh D.I water. Then 0.03 g powder of each synthesized sample was mixed in 200 mL of dye solution followed by sonication and starring in the dark for 1 h to accomplish the adsorption equilibrium.^40^ Next, the prepared dye solution was kept in UV-visible light for 120 min using a xenon arc lamp with a power of 3000 W at room temperature. Furthermore, precise chemicals like *p*-benzoquinone (BQ), isopropyl alcohol (IPA), and ammonium oxalate (AO) were added to the freshly prepared solution as scavengers of superoxide radicals (˙O_2_^−^), holes (h^+^), and hydroxyl radicals (˙OH) respectively participate in dye degradation.

## 3. Results and discussion

**Fig. 1:**
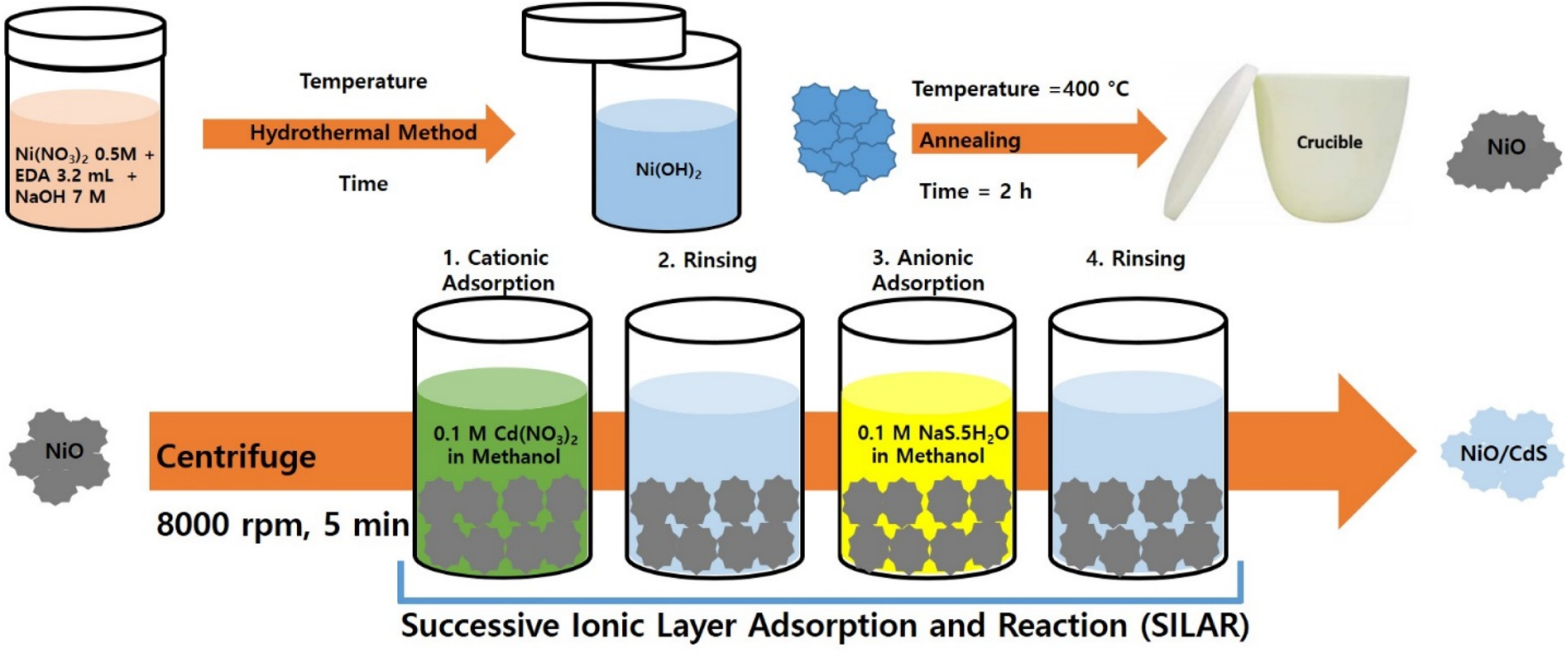
Experimental diagram showing different steps in the synthesis of NiO/CdS p–n heterojunction.

**Fig. 2:**
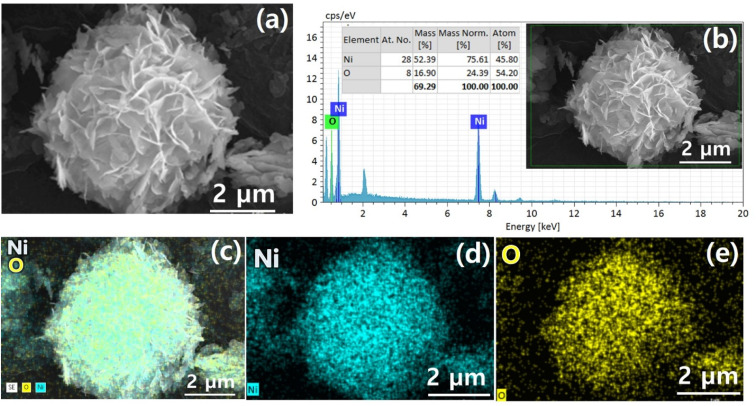
(a) SEM image (b) EDS spectrum, and (c)–(e) elemental area mapping of pristine hierarchical NiO.

**Fig. 3:**
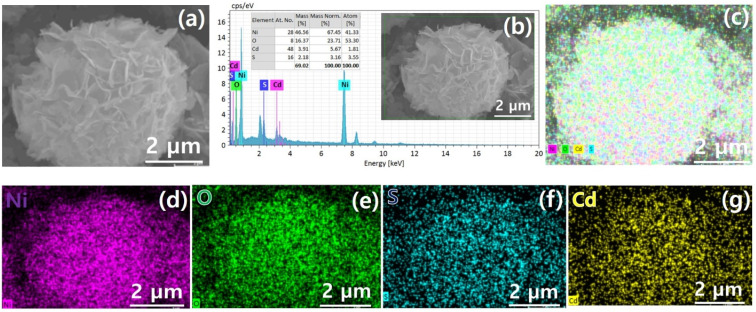
(a) SEM image (b) EDS spectrum and (c)–(g) elemental area mapping of hierarchical NiO/CdS QDS p–n heterojunction.

**Fig. 4:**
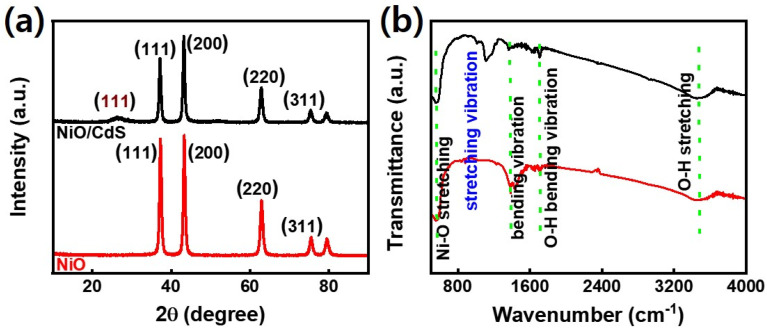
(a) XRD pattern (b) FTIR spectra of NiO as well as NiO/CdS QDs p–n heterojunction.

Fig. 1 demonstrates a graphical illustration of the successful formation of hierarchical NiO as well as NiO/CdS QDs p–n heterojunction. For the successful formation of p–n heterojunction, we have utilized the p-SILAR technique which is facile as well as cost-effective.^13,41^ Utilizing a vacuum oven for effective dryness of the synthesized samples as well as to prevent oxidation. To study the morphology of hierarchical NiO as well as QDs sensitized p–n heterojunction SEM measurements were performed. Pristine NiO demonstrates a nanoflower hierarchical structure as shown in Fig. 2 as well as in Fig. S1.† The SEM image (Fig. 2a) indicates that the structure of pristine NiO is collected from densely packed asymmetrical flakes. DES analysis of the pristine NiO demonstrates 45.80% Ni and 54.20% O matching well with previously reported literature as shown in (Fig. 2b).^2^ Additionally, elemental area mapping was performed to obtain information about the uniform distribution of the constituent elements over the mapped area of pristine NiO as demonstrated in (Fig. 2c–e). Furthermore, SEM images (Fig. 3a) as well as (Fig. S2†) of NiO/CdS possess the same flower-like morphology as demonstrated by pristine hierarchical NiO after deposition of CdS QDs. Additionally, EDS measurements were performed to demonstrate the presence of every constituent element with the atomic ratios as shown in Fig. 3b. EDS analysis data of p–n heterojunction gives the ratio of 41.3% Ni, 53.3% O, 1.8% Cd, and 3.5% S for different elements while Fig. S3† represents elemental atomic percent ratios for Cd/Ni and S/Ni. Additionally, elemental area mapping was executed to obtain information about the uniform distribution of the constituent's element of p–n heterojunction as shown in Fig. 3c–g. This demonstrates the uniform deposition of CdS QDs on hierarchical NiO to form active and ultimate heterojunctions for effective charge transfer. Such an effective and ultimate deposition of CdS QDs on hierarchical NiO to form p–n heterojunction was optimized by PL spectra as shown in Fig. S6,† 6 SILAR cycle of CdS QDs deposited on hierarchical NiO gives lowest PL intensity signifying the energetic charge carrier's separation, migration as well as transport, which boost the photocatalytic efficiency. Furthermore, to study the phase purity of hierarchical NiO, as well as p–n heterojunction XRD measurements, were executed as shown in Fig. 4a as well as (Fig. S4 and S5†). Pristine NiO demonstrates the characteristic peaks at 37°, 43°, 62.9°, and 75° assigned to (111), (200), (220), and (311), demonstrating phase purity of synthesized sample without the formation of any parasitic phases.^42,43^ Furthermore, the XRD pattern of p–n heterojunction shows all the diffraction peaks of NiO along with an additional peak at 26.3° assign to the (111) plane of CdS,^44^ demonstrating the successful formation of p–n heterojunction. We have used the^29,39^ Scherrer equation 
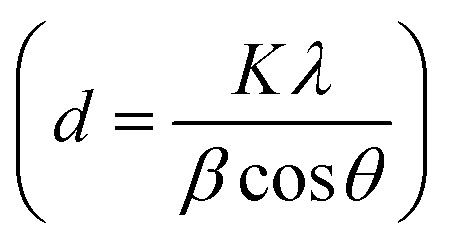
 to estimate the average crystallite size from the broadening of the peaks (instrumental broadening is eliminated). In this equation, *K* dimensionless shape factor generally used as 0.9, *λ* is the wavelength of the X-ray beam, *β* is the line broadening at half maximum intensity and *θ* is the Bragg angle. Our analysis revealed that in our samples the average crystallite size varies between 2.8 (2) nm and 3.0 (2) nm. Furthermore, FTIR measurement was performed to find the bending vibration as well as functional groups attached to the surface of synthesized samples as demonstrated in Fig. 4b. Pristine NiO demonstrates a characteristic peak at ∼573 cm^−1^ assigned to Ni–O stretching vibration mode.^45,46^ Furthermore, a broad absorption band and surface-active groups at ∼3000–3400 cm^−1^ were assigned to the OH stretching mode.^47,48^ Additionally, p–n heterojunction demonstrates all the characteristic peaks of NiO along with characteristic stretching vibration at 1110 cm^−1^ indicating the presence of CdS single.^49,50^

**Fig. 5:**
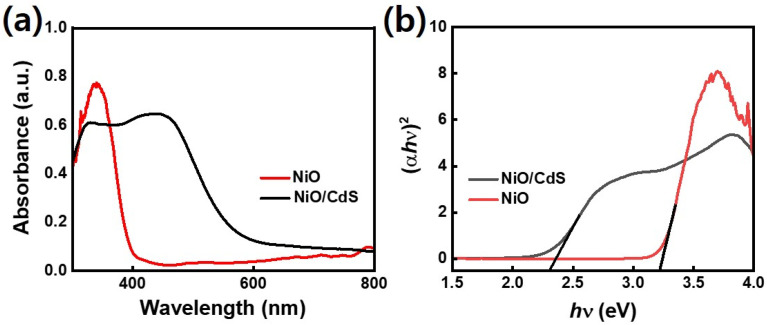
(a) UV-visible absorption (b) Tauc plot of pristine NiO and NiO/CdS.

Furthermore, the optical property of pristine NiO as well as p–n heterojunction was evaluated using UV-vis spectroscopy as shown in Fig. 5a. Compared to pristine NiO, the peak position moved to higher wavelengths for the sample with CdS indicating higher light absorbance which in turn generates a large number of photogenerated charge carriers which is playing a significant role in dye-degradation.^51,52^ Bandgap energy values were extracted for corresponding samples using the equation (*αhν*)^*n*/2^ = *A*(*hν* − *E*_g_) in which *α* represents the absorption coefficient, *h* for Planck's constant, *ν* stand for light frequency, *E*_g_ corresponding bandgap, and A represents a proportionality constant as demonstrated in Fig. 5b. Furthermore, an equation value of *n* is 1 and 4 for direct and indirect bandgap semiconductor respectively. UV-vis spectroscopy lead to a wide bandgap value of 3.3 eV which is in line with the reported bandgap (3.2–4.0 eV) values of NiO.^34^ On the other hand, the spectrum of the NiO/CdS nanocomposite is very interesting: two clear linear slopes are visible in the spectrum indicating strong absorption at 2.4 eV and 3.3 eV.^53^ The lower energy one belongs to NiO/CdS and it leads to a bandgap of 2.4 eV. Such a value of bandgap is also within the reported range of bandgaps for QDs-sensitized hierarchical nanocomposite.^13^

**Fig. 6:**
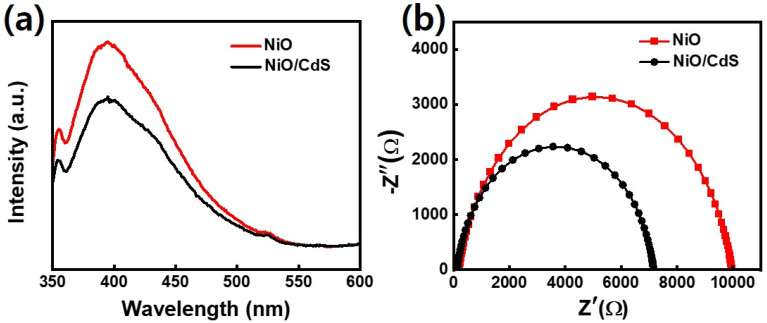
(a) Photoluminescence spectra and (b) Nyquist plots of NiO and NiO/CdS p–n heterojunction.

In semiconducting oxide nanostructures, the PL emission from near band edge (UV) and deep level (DL) defect-related visible regions have been typically observed. In this regard, UV emission is related to the direct recombination of excitons through an exciton–exciton scattering while the visible emission results from the radiative recombination of a photo-generated hole with an electron occupying a defect state like an oxygen vacancy. To study the recombination, separation, and migration mechanism of photogenerated charge carriers in pristine NiO, as well as NiO/CdS QDs p–n heterojunction of all the synthesized samples PL spectra, were executed at an excitation wavelength of 360 nm as shown in Fig. 6a as well as in Fig. S6.† Furthermore, PL spectra of pristine CdS QDs were performed as shown in Fig. S7.† As PL is light emission from any form of matter after the absorption of photons. What why the synthesized samples show a broad UV emission band peaked at 390 nm (≈3.25 eV) and a shoulder peak centered at about 525 nm (≈2.2 eV) in the visible region. The photoluminescence (PL) emission peak intensity is associated with the recombination rate. The emission intensity increases, by increasing the recombination rate of electron/hole pairs. As expected, the PL emission peak intensity of NiO/CdS nanocomposite is lower than the spectrum obtained from bare NiO which indicated the slowed nature of the recombination rate. Such a slow recombination rate in nanocomposite structure is the hallmark of increased photocatalytic efficiency of NiO/CdS structure. Moreover, among different samples, 6 cycles of CdS QDs deposited demonstrate effective charge separation and transport compared to other p-SILAR cycles number. The peak intensity decreases with increasing p-SILAR cycles, which is saturated after 6 cycles as shown in Fig. S6.† This means that 6 cycles are sufficient to suppress the photogenerated charge carriers *via* heterojunction formation for charge separation and transfer to boost photodegradation efficiency. Such effective charge separation, migration, and transfer indicate the strong chemical makeup between the constituent's materials.^29,39^ To further evaluate the charge transfer performance in detail we use the Nyquist plot. The equivalent circuit fitted in the EIS measurement consisted of series resistance (*R*_s_), a charge transfers resistance (*R*_t_) as well as a capacitor (*C*) as shown in Fig. 6b. In Nyquist plots, semicircles are corresponded to (*R*_t_) at the electrolyte/nanomaterial interface. The smaller the semicircle will be the (*R*_t_), while higher will be the electro-catalytic activities.^54–56^ After the deposition of CdS QDs, the diameter of the semicircle became smaller indicating the effective charge transfer as demonstrated in Fig. 6b (the values of *R*_s_, *R*_t,_ and *C* which are given in (Table S1†)). The reported values match well with previously reported literature.^57^

**Fig. 7:**
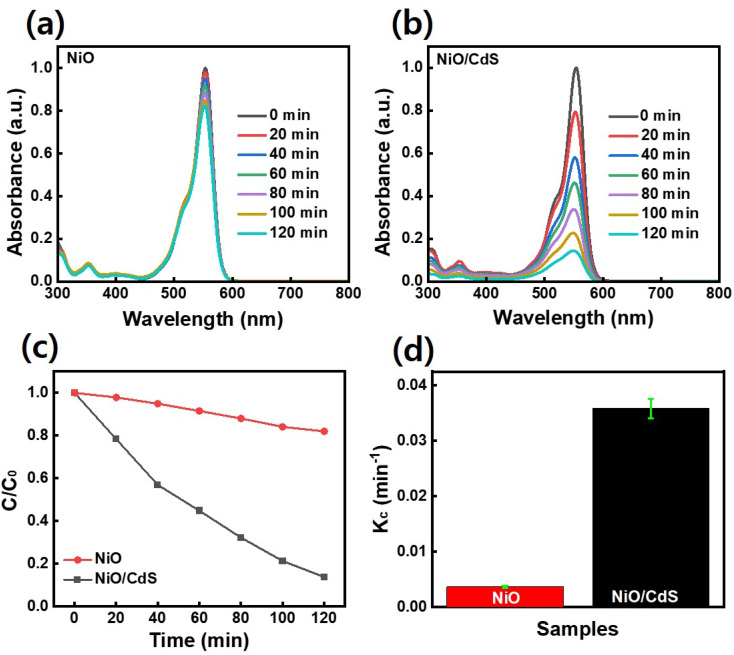
UV-visible light absorbance trend of RhB dye as a function of time of UV-vis irradiation, elucidating UV-visible light induced photocatalytic degradation of RhB in presence of (a) NiO (b) NiO/CdS (c) dye degradation performance (d) reaction rate constant per sample.

To further evaluate the performance of photocatalyst dye-degradation performance, RhB dye was used as shown in Fig. 7. By increasing the irradiation time, the dye concentration and/or peak intensity become less as shown in (Fig. 7a and b). Furthermore, the plot *C*/*C*_0_*vs. t* where *C*_0_ represents the initial concentration and *C* as the concentration of dye after irradiation time (*t*) as demonstrated in Fig. 7c, indicates that a higher concentration change occurs in the case of QDs sensitized photocatalyst compare to pristine NiO. The QDs sensitized photocatalysts demonstrate a higher reaction rate constant (*K*_c_) value (Fig. 7d) compared to pristine NiO, under the same temperature and pressure condition while degrading 86.5% RhB dye in 2 h compared to pristine NiO which degrade 18%.

**Fig. 8:**
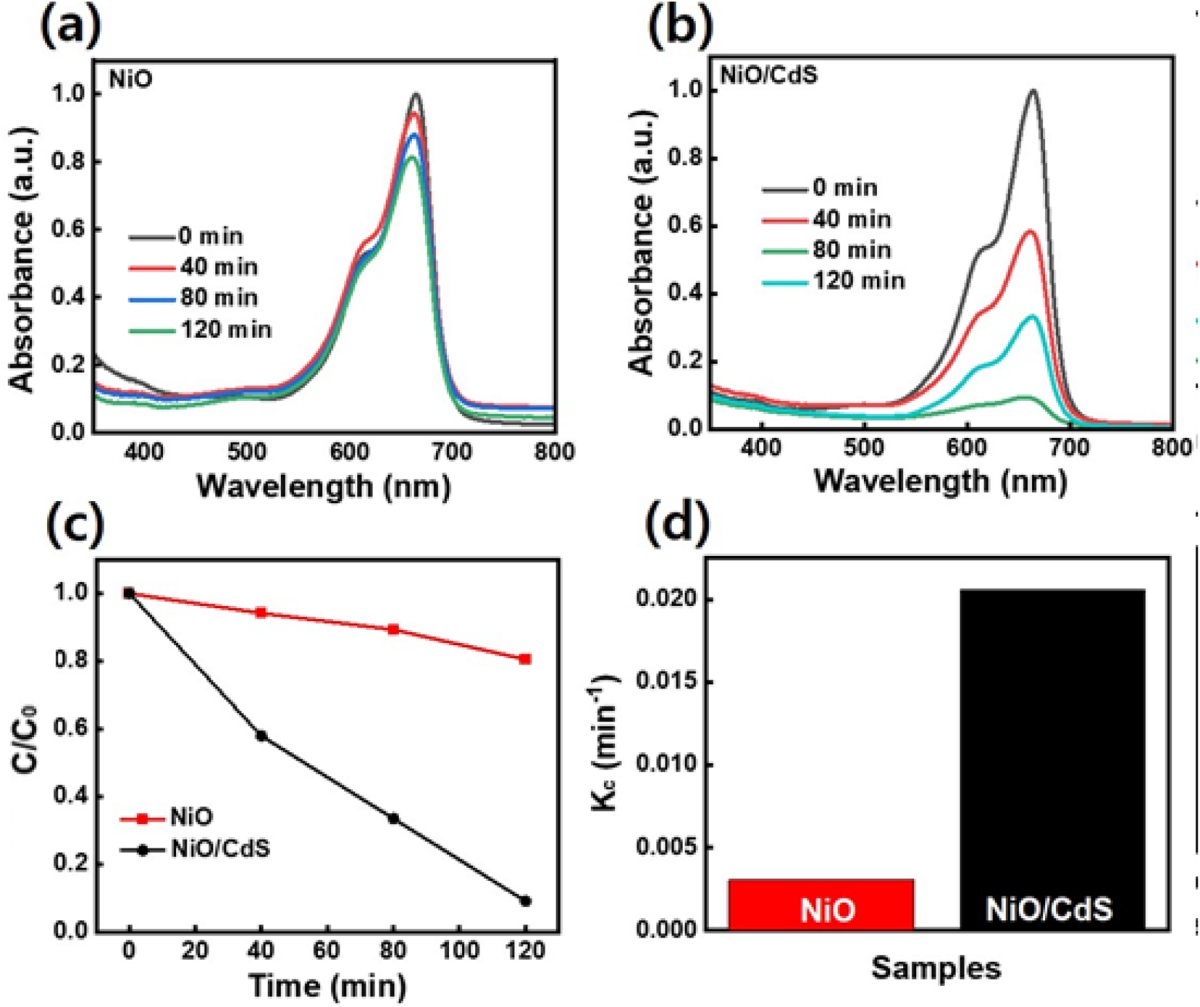
UV-visible light absorbance trend of MB dye as a function of time of UV-vis irradiation, elucidating UV-visible light induced photocatalytic degradation of MB in presence of (a) NiO (b) NiO/CdS (c) dye degradation performance (d) reaction rate constant.

The performance of pristine NiO and QDs sensitized photocatalyst dye-degradation for MB dye were also examined and shown in Fig. 8. By increasing the irradiation time, the dye concentration and/or peak intensity is reduced as shown in (Fig. 8a and b). Furthermore, the plot *C*/*C*_0_*vs. t* as demonstrated in Fig. 8c, indicates that a higher concentration change occurs in the case of QDs-sensitized photocatalyst compare to pristine NiO. The QDs-sensitized photocatalyst demonstrates a higher *K*_c_ value (Fig. 8d) compared to pristine NiO, under the same temperature and pressure condition while degrading 90.9% MB dye in 2 h compared to pristine NiO which degrade 20.1%. Furthermore, scavenging experiments under UV-visible irradiation were executed to evaluate the role of ˙O_2_^−^, H^+^, and ˙OH, respectively in dye-degradation.

**Fig. 9:**
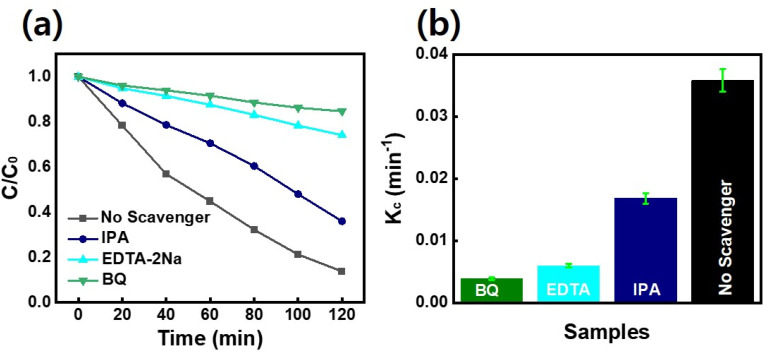
(a) Results of dye degradation experiments carried out under the presence of scavengers for optimal NiO/CdS p–n heterojunction (b) variation in *K*_c_.

**Fig. 10:**
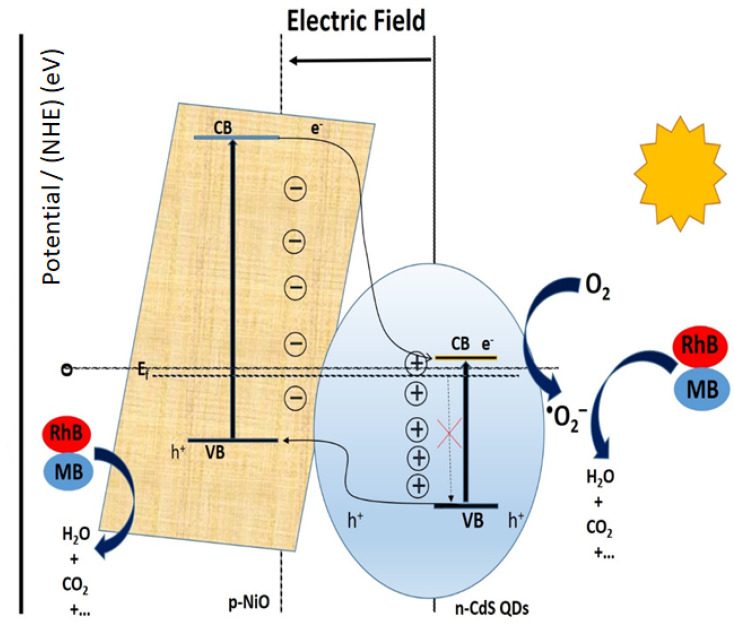
Schematic representation of photogenerated charge transfer in NiO/CdS p–n heterojunction.

Fig. 9a and b demonstrates that the addition of scavengers such as BQ and AO greatly suppressed the photodegradation process.^2^ Additionally, IPA slightly affects the photodegradation rate. The above scavenger experiment ascertains that h^+^ and ˙O_2_^−^ are key reactive species in dye degradation. A schematic was constructed which illustrates the successful transformation of charge carriers between p–n heterojunction for dye degradation on a scavenger data basis as demonstrated in Fig. 10.^2^ Intrinsically, Fermi levels are positioned near CB and VB positions for n and p-type semiconductors, respectively.^58^ In particular, the Fermi energy for NiO is about 5 eV, while for CdS it is 0.1 eV below the conduction and energy level of 4.9 eV. Upon the p–n heterojunction formation, their corresponding Fermi energy level could be aligned under thermodynamic equilibrium; consequently, it results in the shifting in energy band level and intrinsic electric field formation at the semiconductor interfaces.^33,58^ Thus, the formation of an internal electric field effectively separates the photogenerated charge carriers which are also thermodynamically favorable. It has been seen that the diffusion of electron–hole near the p–n junction interface happens until the Fermi-level equilibrium which creates ‘‘charged” space or the so-called internal electric field.^33^ Furthermore, upon light irradiation, photogenerated electrons jump to the CB of CdS as well as NiO. Furthermore, excited electron resides on the CB of NiO and also transfer to the CB of CdS QDs due to thermodynamically favorable band gap position. The excited electrons residing on CB of CdS convert the absorbed O_2_ effectively to active ˙O_2_^−^ radical which plays a significant role in dye-degradation. Moreover, due to the internal electric field, the photo-induced holes are thermodynamically favorable to transfer from VB of CdS to VB of NiO. This special technique effectively minimizes the recombination rate which boosted dye-degradation performance.^59^ Main oxidation/reduction reactions take place due to (˙O_2_^−^ and h^+^) species which effectively decompose the RhB as well as MB dye molecules. As a result of light irradiation, the following reaction takes place eqn (2):

1 NiO/CdS + *hv* → CdS (h^+^ + e^−^)/NiO

2 CdS (h^+^ + e^−^)/NiO → CdS (e_CB_^−^)/NiO (h_VB_^+^)

3 CdS (e_CB_^−^) + O_2_ → ˙O_2_^−^

4 MB/RhB + ˙O_2_^−^ → degradation product

5 MB/RhB + NiO (h_VB_^+^) → degradation product

**Fig. 11:**
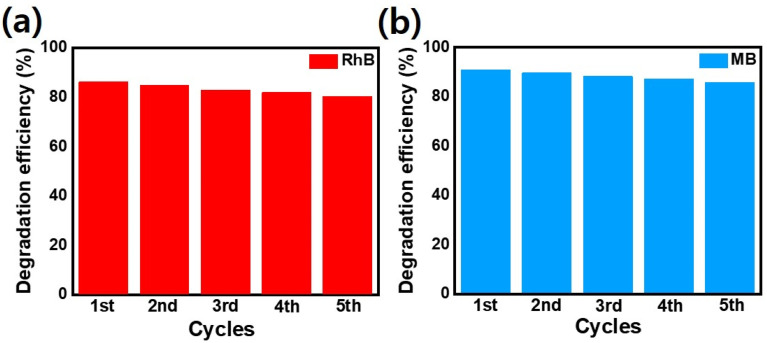
Stability test of NiO/CdS nanocomposite using (a) RhB as well as (b) MB dyes.

The efficient photogenerated charge separation in synthesized p–n heterojunction matches well with the previously reported literature.^26^ Additionally, the photo as well as chemical stability, selectivity, and reusability of synthesized nanocomposite play a crucial role in dye-degradation performance.^60–63^Fig. 11 indicates that after 5 consecutive cycles, very small photocatalytic performance changes were observed for p–n heterojunction demonstrating the stability as well as reusability of the photocatalyst. An interesting feature of the degradation process is the response of the material to MB and RhB dyes: both decayed similarly. Main oxidation/reduction reactions take place due to (˙O_2_^−^ and h^+^) species which effectively decompose the RhB as well as MB dye molecules. Note that such small differences in the performance of the MB and RhB dyes were also observed by Hoseini. *et al.*^64^ on the n-type CdS nanorods/p-type LaFeO_3_ heterojunction nanocomposite. We believe such behavior is associated with CdS and its enhanced ability to convert the absorbed O_2_ effectively to active ˙O_2_^−^ radicals.

## 4. Summary

In summary, in the present work, p–n heterojunctions were realized by depositing an optimum amount of CdS QDs on nanoflower hierarchical NiO *via* the p-SILAR technique. Detailed microstructural analysis based on SEM/EDX and XRD revealed the successful and high-quality formation of NiO and CdS nanostructures and more importantly NiO/CdS nanocomposites. XRD analysis revealed that in our samples the average crystallite size of CdS quantum dots varies between 2.8 (2) nm and 3.0 (2) nm. Spectroscopic techniques such as photoluminescence, FTIR, and UV-vis were used to evaluate surface chemistry and electronic processes related to the p–n heterojunction formation. Moreover, the photocatalytic performance of the synthesized structures was evaluated using RhB dye. The synthesized photocatalyst demonstrates enhanced dye degradation performance due to (i) a large surface area (ii) a large number of unsaturated oxygen vacancies, and (iii) an optimized number of p–n heterojunctions which effectively separate and transfer the photogenerated charge carriers. In comparison to the pristine NiO, the p–n heterojunction photocatalyst demonstrate enhanced dye-degradation performance.

## Author contributions

Junaid Khan: investigation, writing – original draft and formal analysis, Gohar Ali: sofware, writing-original draft, writing – review & editing and methodology, supervision, Ayesha Samreen: conceptualization, formal analysis, writing – review & editing, Shahbaz Ahmad: writing – review & editing, Sarfraz Ahmad: software, writing – review & editing, Mehmet Egilmez: conceptualization, writing – original draft, formal analysis, writing – review & editing and methodology, data curation, supervision, Sadiq Amin; investigation, writing – review & editing, Nadia Khan: investigation, writing – review & editing.

## Conflicts of interest

There are no conflicts of interest to declare.

## Supplementary Material

RA-012-D2RA05657G-s001
