# Supplementary material for: Quantum-dot sensitized hierarchical NiO p–n heterojunction for effective photocatalytic performance
Source: RSC Adv. 2022 Nov 11;12(50):32459–70. doi: 10.1039/d2ra05657g (PMC9651132; doi:10.1039/d2ra05657g)
Supplement: RA-012-D2RA05657G-s001 [file RA-012-D2RA05657G-s001.pdf]

Electronic Supplementary Material (ESI) for RSC Advances.
This journal is © The Royal Society of Chemistry 2022

Quantum-dot Sensitized hierarchical NiO p-n heterojunction for effective photocatalytic performance

Junaid Khan[1], Gohar Ali[2], Ayesha Samreen[1], Shahbaz Ahmad [3,4], Sarfraz Ahmad[5], Mehmet Egilmez[3,4*], Sadiq Amin[6], Nadia Khan[7]

[1]*Department of Physics, University of Peshawar, Peshawar, Pakistan*

[2] *Department of Materials Science and Chemical Engineering, Hanyang University, Ansan,15588, Republic of Korea*

[3] *Department of Physics, American University of Sharjah, Sharjah, United Arab Emirates*

[4] *Materials Science and Engineering Program, College of Arts and Sciences, American University of Sharjah, Sharjah, United Arab Emirates*

[5] *Department of Mathematics, Abbottabad University of Science and Technology, Abbottabad, 22500, Pakistan*

[6] *Material Research Laboratory, Department of Physics, University of Peshawar 25120, Pakistan7[g] Department of Physics, Khushal Khan Khattak University, Karak 27200, Khyber-Pakhtunkhwa Pakistan*

*Corresponding author: Mehmet Egilmez, E-mail: megilmez@aus.edu

Keywords: Hierarchical NiO, CdS QDs, p-n Heterojunction; Dye-degradation

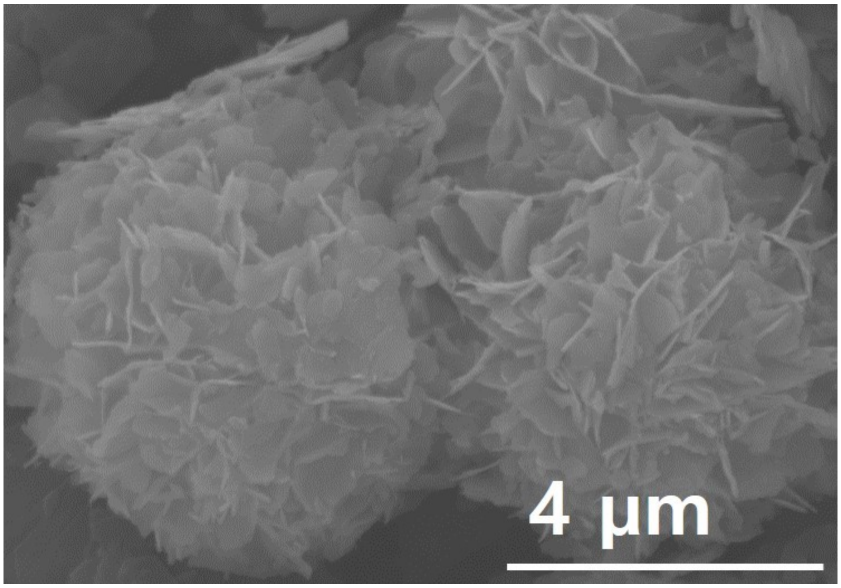

Fig. S1: (a) Scanning electron microscopic image of pristine hierarchical NiO.

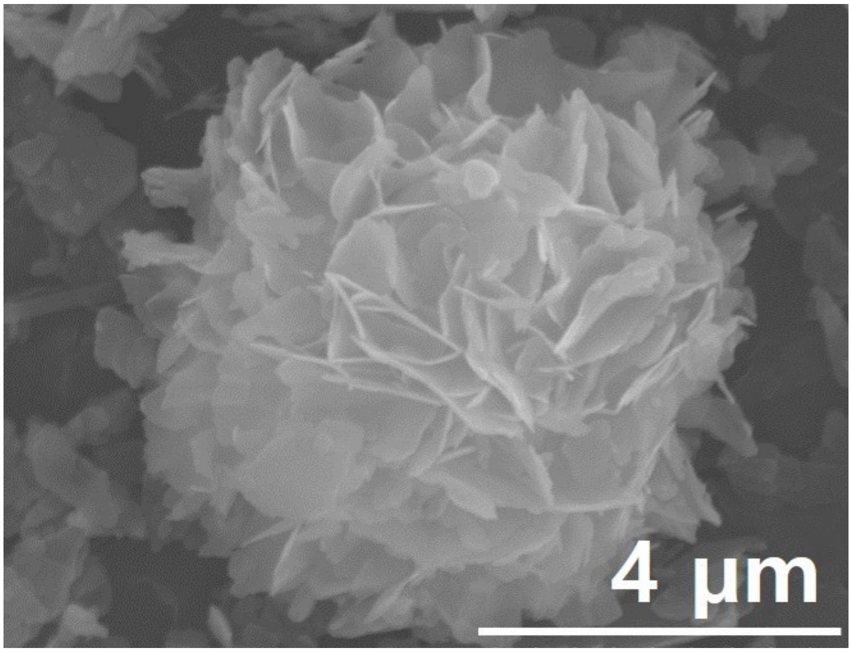

Fig. S2: (a) Scanning electron microscopic image of hierarchical NiO/CdS heterojunction.

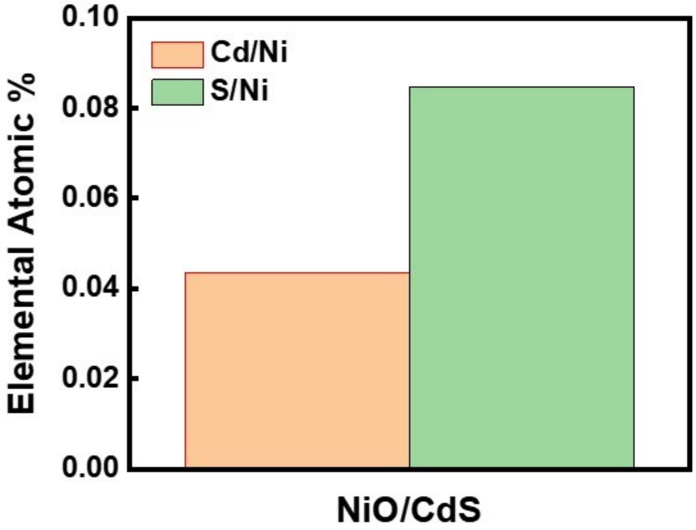

Fig. S3 : EDS-based quantitative comparison for Cd/Ni and S/Ni for CdS/NiO by atomic percent.

NiO_CdS

#	Phase ID	Chemical Formula	PDF-#	RIR	FOM (n)	Scale(I)	Shift(x)	Space Group	a (Å)	b (Å)	c (Å)
1	Bunsenite	*NiO*	98-000-0133	5.19	0.9 (05)	0.940	0.020°	cFm3m (225)	4.1780	4.1780	4.1780

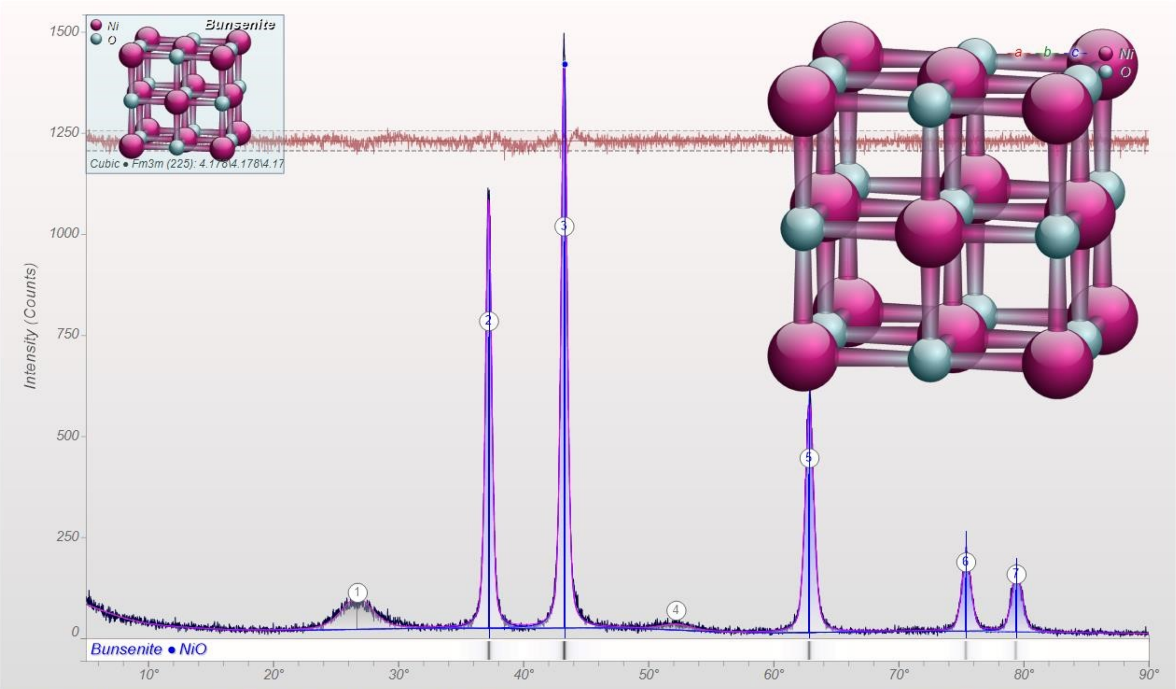

[D:\data\hanyang\PTJ\20210610\NiO_CdS.raw] 5.0°/90.0°/0.02°/0.3(s), I(p)=1498.0/4.0, Cu(40kV,100mA)

Thursday, June 10, 2021, 2:38 PM ● Hanyang University

NiO_CdS

#	Angle(°)	d(Å)	Centroid	Centroid	BG	Height	H/σ	Area(α1)	Area(α1)%	A/σ	S/L(1.0)	Shape¹	FWHM(°)	XS(Å)	(h k l)	Pha
1	26.687 (0.018)	3.3376 (0.0045)	26.687°	3.3376Å	23.0	52.0 (0.6)	84.9	194.7 (2.9)	28.1 (0.4)%	66.4	0.0283	0.488v	3.000 (0.022)	27		
2	37.186 (0.002)	2.4159 (0.0002)	37.186°	2.4159Å	25.5	721.2 (4.0)	179.9	514.9 (3.7)	74.3 (0.5)%	139.0	0.0283	0.488v	0.559 (0.002)	158	(1 1 1)	Buns
3	43.217 (0.001)	2.0917 (0.0001)	43.217°	2.0917Å	26.4	954.7 (4.5)	210.3	692.9 (4.3)	100.0 (0.6)%	159.3	0.0283	0.488v	0.570 (0.002)	158	(2 0 0)	Buns
4	52.168 (0.067)	1.7519 (0.0042)	52.168°	1.7519Å	21.3	9.6 (0.4)	21.3	33.1 (2.0)	4.8 (0.3)%	16.3	0.0283	0.488v	2.794 (0.083)	32		
5	62.810 (0.003)	1.4783 (0.0001)	62.810°	1.4783Å	15.5	392.6 (2.5)	156.1	389.0 (3.2)	56.1 (0.5)%	120.8	0.0283	0.488v	0.784 (0.003)	123	(2 2 0)	Buns
6	75.340 (0.005)	1.2605 (0.0002)	75.340°	1.2605Å	18.3	132.1 (1.6)	84.6	137.5 (2.1)	19.8 (0.3)%	64.5	0.0283	0.488v	0.827 (0.006)	125	(3 1 1)	Buns
7	79.364 (0.007)	1.2064 (0.0002)	79.364°	1.2064Å	16.8	103.7 (1.3)	78.3	121.5 (2.0)	17.5 (0.3)%	60.0	0.0283	0.488v	0.934 (0.008)	113	(2 2 2)	Buns

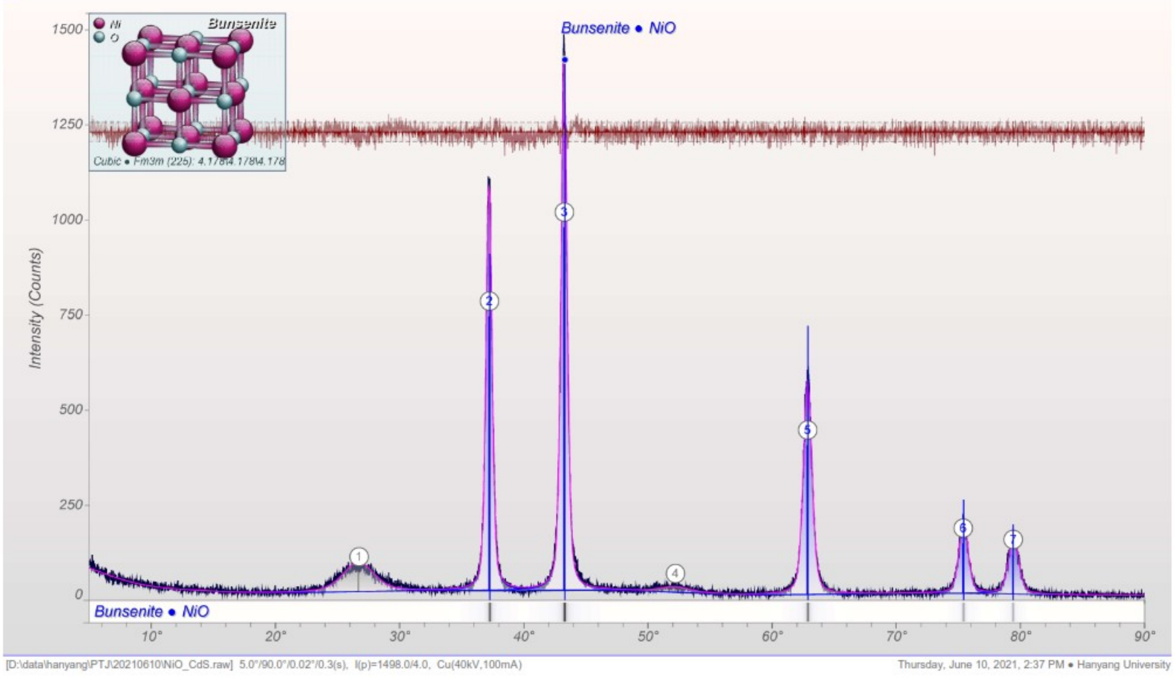

Fig. S4: XRD pattern NiO-CdS

NiO

#	Phase ID	Chemical Formula	PDF-#	RIR	FOM (n)	Scale(I)	Shift(x)	Space Group	a (Å)	b (Å)	c (Å)
1	Bunsenite	*NiO*	98-000-0133	5.19	1.0 (05)	1.000	0.080°	cFm3m (225)	4.1780	4.1780	4.1780

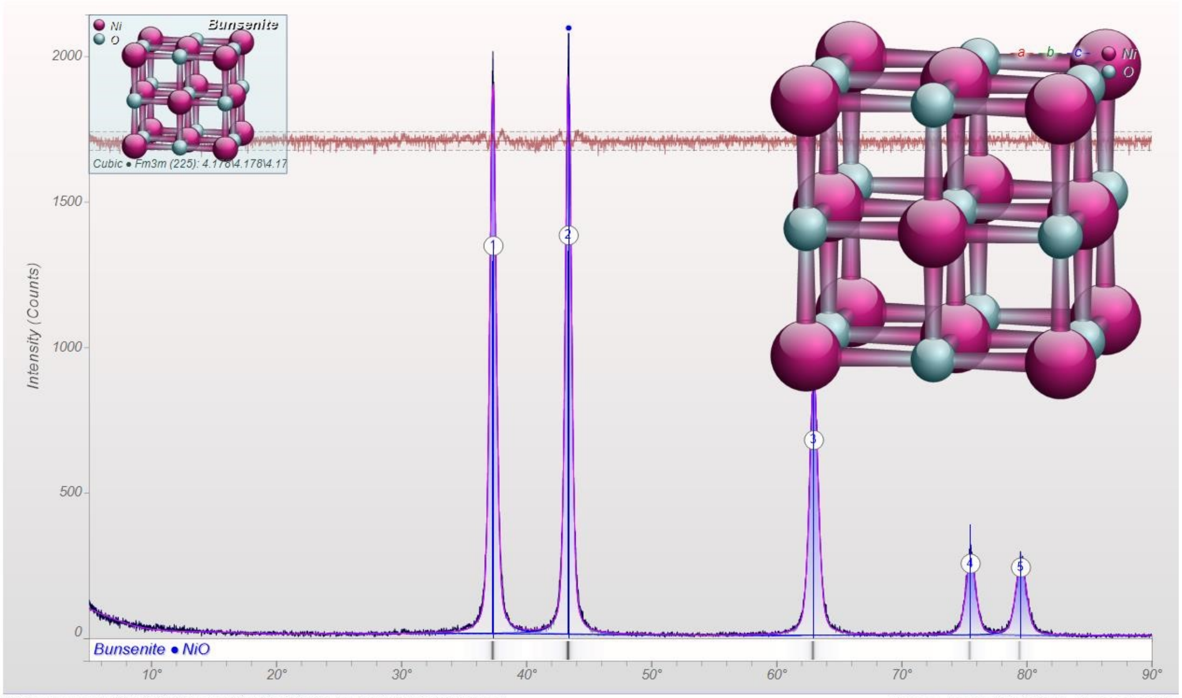

[D:\data\hanyang\PTJ\20210610\NiO.raw] 5.0°/90.0°/0.02°/0.3(s), I(p)=2081.0/2.0, Cu(40kV,100mA) Thursday, June 10, 2021, 1:56 PM • Hanyang University

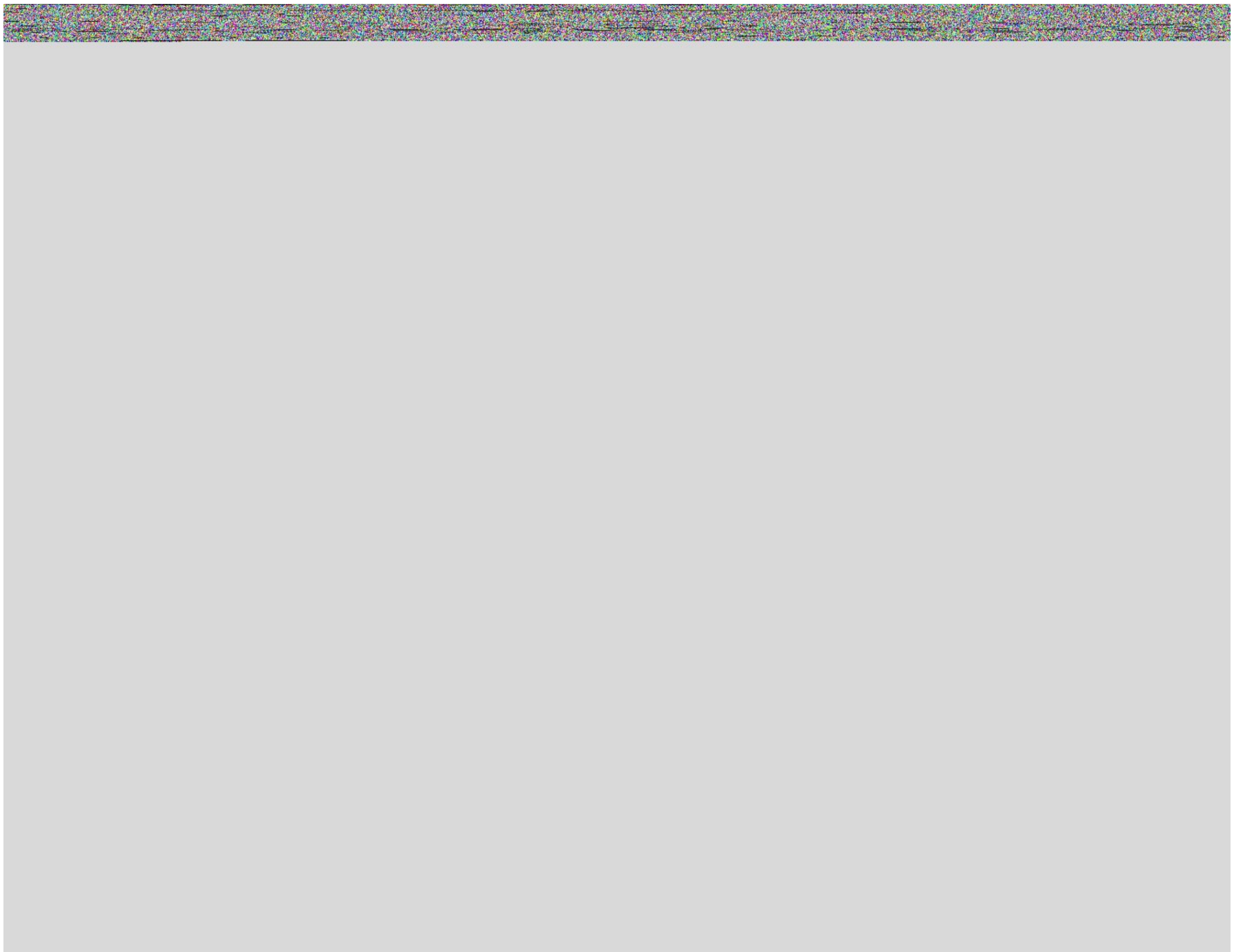

Fig. S5: XRD pattern NiO-CdS.

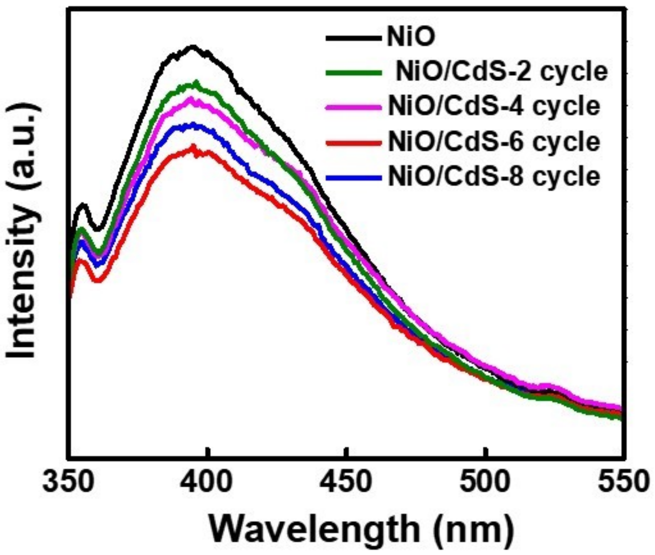

Fig. S6: Photoluminescence spectra of pristine NiO as well as different NiO/CdS p-n heterojunctions.

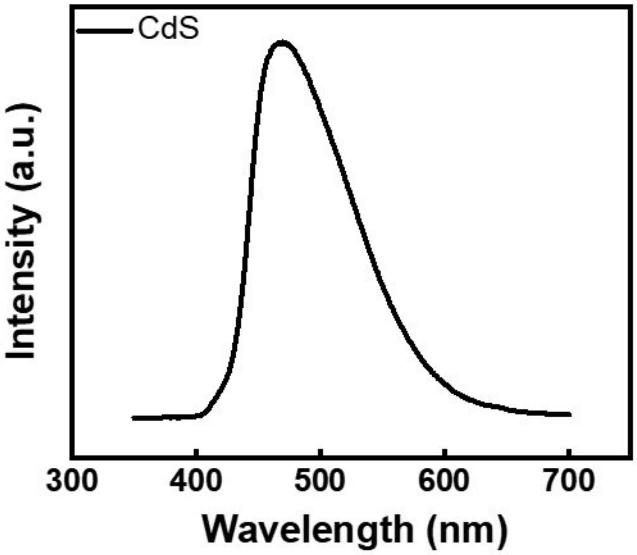

Fig. S7: Photoluminescence spectra of pristine CdS QDs.

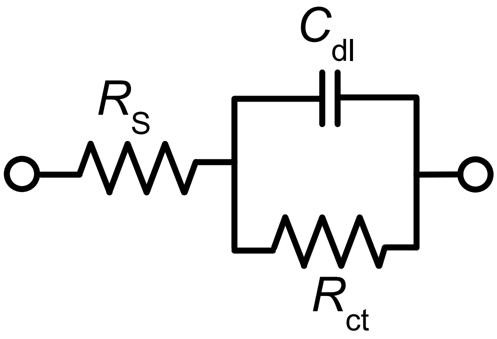

Fig. S8: Equivalent circuit of EIS data.

Table S1: Various electrochemical parameters derived from the EIS study.

Photocatalysts	R_s (Ω)	R_{ct} (Ω)	C_{dl} (μF)
**NiO**	6.22	9989	0.733
**NiO/CdS**	4.33	7149	0.551
